# Design and evaluation of a power tiller vegetable seedling transplanter with dibbler and furrow type

**DOI:** 10.1016/j.heliyon.2023.e17827

**Published:** 2023-07-08

**Authors:** Md Sumon Miah, Md Mashiur Rahman, Muhammad Arshadul Hoque, Sobhy M. Ibrahim, Muhammad Sultan, Redmond R. Shamshiri, Mustafa Ucgul, Mahedi Hasan, Tasneem Nahar Barna

**Affiliations:** a Farm Machinery and Postharvest Process Engineering Division, Bangladesh Agricultural Research Institute, Joydebpur, Gazipur, 1701, Bangladesh; b Agricultural Engineering Division, Pulses Research Center & Regional Agricultural Research Station, Bangladesh Agricultural Research Institute, Ishurdi, 6620, Pabna, Bangladesh; c Department of Biochemistry, College of Science, King Saud University, P.O. Box 2455, Riyadh, 11451, Saudi Arabia; d Department of Agricultural Engineering, Bahauddin Zakariya University, Multan, 60800, Pakistan; e Department of Engineering for Crop Production, Leibniz Institute for Agricultural Engineering and Bioeconomy, 14469, Potsdam, Germany; f Faculty of Science and Engineering, Southern Cross University, Lismore, NSW, 2480, Australia

**Keywords:** Computational design analysis, Mechanized transplanter, Semi-automatic vegetable transplanter, Seedling planting, Vegetable farming

## Abstract

Vegetable production plays a vital role in ensuring food security in Bangladesh. However, the majority of vegetable seedlings are currently transplanted manually, which is not only time-consuming but also labor-intensive and costly. In this context, a semi-automated transplanter can be considered as an alternative solution for mechanized seedling transplanting. To mechanize seedling operations, two types of transplanters were designed, fabricated and tested: the power tiller-operated semi-automatic dibbler vegetable seedling (DVS) transplanter and the furrow opener vegetable seedling (FVS) transplanter. The goal was to evaluate their performance and impact on field crop productivity. In the DVS transplanter design, the larger sprocket was adjusted to enhance the precision of hole-making by pressing the dibbler into the soil, creating holes where seedlings would be transplanted. On the other hand, the FVS transplanter utilized a furrow opener to create furrows, and the seedling is placed in these furrow at a specific distance from the furrow opener wall, where the distance between seedlings within the furrow could be adjusted based on the specific requirements of the seedling crop. The results of the evaluation indicated that both transplanters successfully planted seedlings without any missing placements, while hole covering was achieved at 115 and 118.2% for the DVS and FVS transplanters, respectively. The field capacity and field efficiency for both transplanters were determined to be 0.05 ha h^−1^ and 61.18%, respectively, with a coefficient of variation of 5% or less. Field tests conducted with brinjal crops at a forward speed of 1.2 km h^−1^ and a spacing of 0.7 × 0.6 m demonstrated that both designs yielded higher yield productivity compared to manual transplantation. Additionally, no issues related to vegetative development were observed. Both transplanters exhibited promising performance and significant potential in terms of accurately transplanting seedlings, and ensuring satisfactory transplantation quality. Furthermore, these transplanters offer several advantages, including less time-consuming, lower labor demands and even distribution of seedlings. This design encourages small to medium-level farmers seeking to engage in mechanized vegetable farming practices.

## 1 Introduction

Bangladesh is a predominantly agricultural country, and agriculture plays a significant part in the national economy. Agricultural production systems have intensified through mechanized agriculture methods in recent decades [1], and significant progress has been made in vegetable production and export. In the country, more than 60 species of indigenous and exotic vegetables are grown [2]. Currently, 31.3 million metric tons of vegetables are grown on 3.74 million hectares [2,3]. According to the FAO report, vegetable production has expanded to reach 1.97 crore tonnes, with a sevenfold increase in the last 12 years [4], and Bangladesh is ranked third in the world for vegetable production, behind China and India. In the last decade, the amount of land under vegetable cultivation in the country has increased by 5%. Over the previous three years, vegetable production has grown by 6%. At the moment, just 2.57% of Bangladesh's total land area is allocated to the cultivation of vegetables [5].

Most vegetables are grown in Bangladesh in dryland soils with raised bed systems. Bare root seedlings transplanted manually from nursery beds at the necessary spacings are arranged in rows in raised beds. After that, the soil is spread and compacted around the seedling (using a spade). Transplanting of commonly grown vegetables such as eggplant, onion, brinjal, or chili pepper requires 185–260 man-hours per hectare [6]. In some low-mechanization farms, instead of raised beds, seedbeds are prepared, and seedlings are transplanted physically in rows; this process is called flat planting. The vast majority of vegetable seedlings are transplanted by hand, which requires creating a hole in the soil, positioning the seedlings at the appropriate depth, and then pressing the soil back into the hole. This approach requires around 320 man-hours per hectare [7]. In particular, manual seedling transplantation is time-consuming, requires high labor, and occasionally leads to uneven plant distribution [[8], [9], [10]]. Sixty percent of vegetables are grown during winter [11], making it more challenging due to the lack of manpower. Furthermore, for the double-row seedling planting in one ridge (which is a traditional practice), it would be challenging to perform harvest of the previous crop, substrate preparation, and transplantation all in a short amount of time. Therefore, to reduce labor costs and increase the quality of transplanting and crop production, the mechanization of the transplanting operation is essential.

To date, very little study has been performed to develop a power tiller operated vegetable transplanter that is both affordable and effective other than a tractor mounted transplanter for precise planting of seedlings into the subsoil at small to medium size vegetable farming in developing countries. An overview of the various mechanized seedling transplanters has proposed by researchers provided in Table 1, which may help one gain a better understanding of the developed and present seedling transplanters.

**Table 1:** Characteristics of the different seedling transplanters by considering various design parameters.

SL No.	Transplanter Type	Power Sources	Mechanized types	Design Parameters	Results	References
1	Power tiller operated vegetable transplanter	Power tiller	Semi-automatic	Inclined press wheels attachment for transplanting on flat bed, and the mould board with truncated conical ridge shaper attachment	Effective field capacity was found to be 0.057, 0.058, 0.073, 0.046 and 0.074 ha h^−1^ for transplanting cabbage, chilli, tomato, knolkhol and brinjal, respectively	[12]
2	Tractor mounted vegetable transplanter	Tractor	Semi- automatic	Conical distributor cup into the fall tube and transferred to the furrow formed by the furrower	Field experiment was conducted at the three levels of forward speed, and two levels of cultivation depth at 5 and 10 cm	[13]
3	Walk-behind hand tractor two-row vegetable transplanter	Power tiller	Automatic	Horizontal chain conveyor to a hopper-type planting device	Planting rate of the transplanter was 31 plants min^−1^ and the field capacity was 0.045 ha h^−1^ for transplanting tomato and chilli pepper	[14]
4	Tractor mounted automatic vegetable transplanter	Tractor	Automatic	Rotating finger device with a push-type mechanism for plug seedlings	Field transplanting efficiency and effective field capacity was 76.12% and 0.093 ha h^−1^, respectively	[15]
5	Power tiller operated paddy transplanter	Power tiller	Semi-automatic	Modification of the planting claw	Increase in spacing resulted in more tillers and more panicle per plant, increase in yield with an increase in spacing	[16]
6	Power tiller operated seedling vegetable transplanter (this study)	Power tiller	Semi-automatic	Dibbler and furrow opener used to transplant seedlings		–

Mechanization of transplanting refers to the decrease in manpower requirements in cultivating operations that give the least amount of seedling damage and the highest level of cultivating efficiency [12]. Although several mechanized transplanters are available in the market, they are costly and require additional power consumption [[13], [17], [18], [19]]. For countries like Bangladesh, mechanized planters should be affordable so local farmers can widely adopt them. Currently, Bangladesh has 700,000 active power tillers, a two-wheeled implement fitted with rotary tillers, and around 50,000 tillers imported annually [20]. Consequently, there has been plenty of opportunity to draw and use these power tillers to operate a useable power tiller-operated seedling transplanter for transplanting vegetable seedlings. For this reason, an alternate seedling transplanting technology may be a semi-automatic power tiller operated transplanter.

Currently, efficient semi-automatic vegetable seedling transplanter for developing countries is not available at small to medium level vegetable farms. Designing a cost-efficient transplanter that uses the same energy source as a power tiller can significantly help the local vegetable farming industry. However, traditional machine design methods are unable to effectively improve the structure's safety. Additionally, traditional methods of machine design result in incalculable production costs and structural weights. Therefore, a computational design analysis has been introduced to this machine design in order to reduce manufacturing costs, time, difficulty, and weight, but also with the appropriate material to ensure the body structure's stiffness, strength, and stability [21,22]. Moreover, analyzing adopting the finite element method (FEM) could also be introduced in this study in order to analyze different difficulties and after receiving results from computer simulation, the research might then be carried out to experimental testing design [22,23]. Therefore, this research aims to design and develop semi-automatic power tiller-operated vegetable transplanters for vegetable seedlings transplanting using the Solidwork™ software, that can be attached to the power tiller after removing the rotavator tillage attachment. After that, the performance assessment of the novel constructed transplanter was then evaluated in the laboratory as well as on the experimental field, and the results were compared to the manual transplanter methods.

## 2 Materials and methods

### 2.1 Design considerations

The vegetable seedling transplanter was carefully designed, taking into account several factors to ensure optimal performance. These factors include ease of construction, ease of adjustment, seedling dropping mechanism, operational maneuverability of transplanter and seedling transplanting mechanism. In addition to this, the proposed transplanter offer the advantage of transplanting seedlings in two rows simultaneously with just one pass. Moreover, the seedling transplanter was constructed to meet some design requirements includings (i) capability to transplant different types of seedlings, (ii) flexibility to accommodate different planting modes, such as dibbler or furrow opener, and (iii) adjustable spacing and depth of seedling transplanting. It is worth noting that the forward speed of the machine was designed to align with the walking speed typically observed in many agricultural machines. These design requirements were evaluated through computational design analysis and considered in conjunction with the cultural practices for vegetable seedling transplanting in Bangladesh.

### 2.2 Computational design analysis

**Fig. 1:**
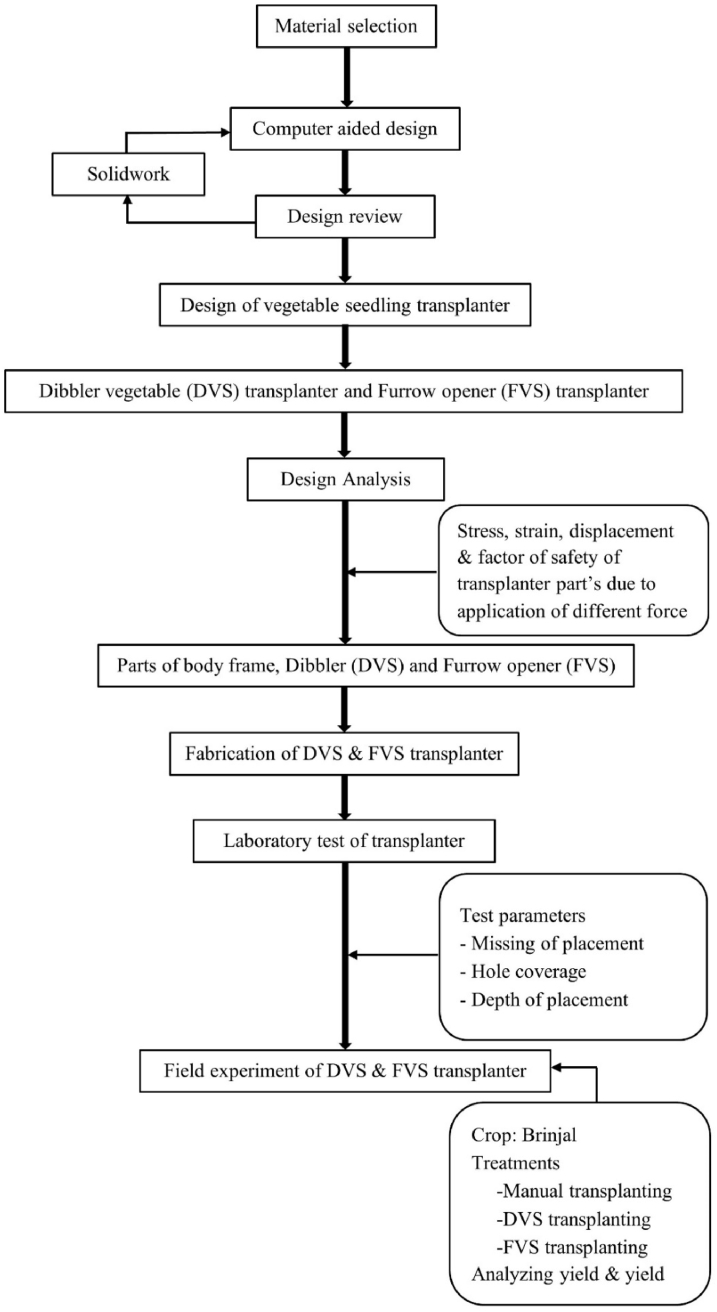
The design process flow chart for this study.

**Fig. 2:**
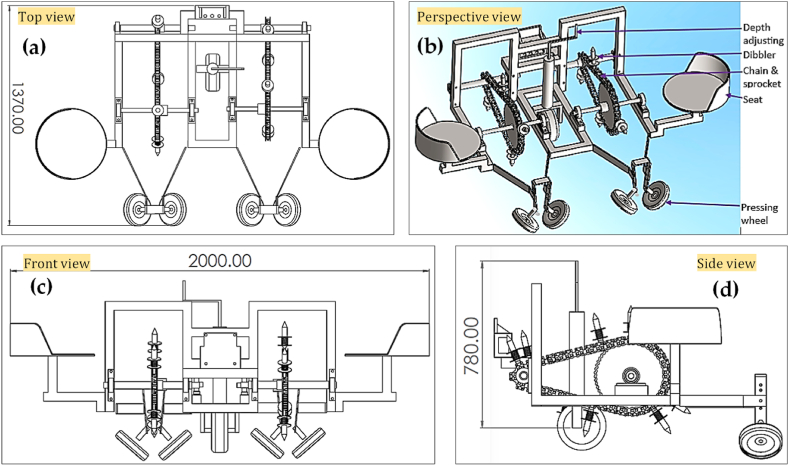
The schematic views of the dibbler vegetable seedling (DVS) transplanter: (a) top view; (b) perspective view; (c) front view; and (d) side view. All dimensions are in mm.

**Fig. 3:**
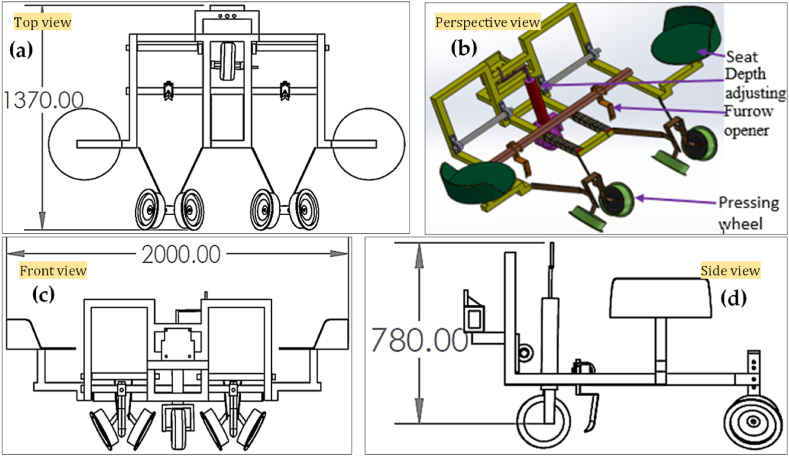
Different views of the furrow opener vegetable seedling (FVS) transplanter: (a) top view; (b) perspective view; (c) front view; and (d) side view. All dimensions are in mm.

The flow chart for the design process is shown in Fig. 1. The Solidwork 2018™ software was employed in the computational design process. A design analysis was conducted on the developed seedling transplanters to enhance their usability and increase field capacity. The three-dimensional computer aided design (3D-CAD) modeling of a DVS and FVS transplanter is shown in Figs. 2 and 3, respectively. The precise computer-aided design (CAD) of the transplanter was designed with the intention of placing seedlings without any being missed; for the areas of the transplanters where the primary force acts. A static force and strength analysis were carried out with Solidwork simulation/Computer-aided engineering (CAE) tools. It is necessary to ensure the right factor of safety, stress and strain of materials in the simulation process when used to accommodate the various load imposed on the materials. For this, the Huber-Mises-Hencky's theory was adopted in this analysis, which is an indicator for measuring the material failure in a uniaxial tensile test [24,25]. In the current model for the computer simulation, the assumption about the materials is that they are homogenous, isotropic, and linearly elastic [[26], [27], [28]]. The most critical parts of the transplanters, such as the furrow opener, dibbler, and frame, were analyzed by SolidWorks simulation to calculate and reduce the stress, strain, displacement, and factor of safety using Huber-Mises-Hencky's theory [19,24]. Also, the geometric model and other related design parameters are considered to address the stress, strain, displacement and factor of safety of different parts in the design analysis, as these parameters used in many mechanical components would impact to machine's overall performance [[29], [30], [31]].

### 2.3 Description and structural design of vegetable seedling transplanter

**Fig. 4:**
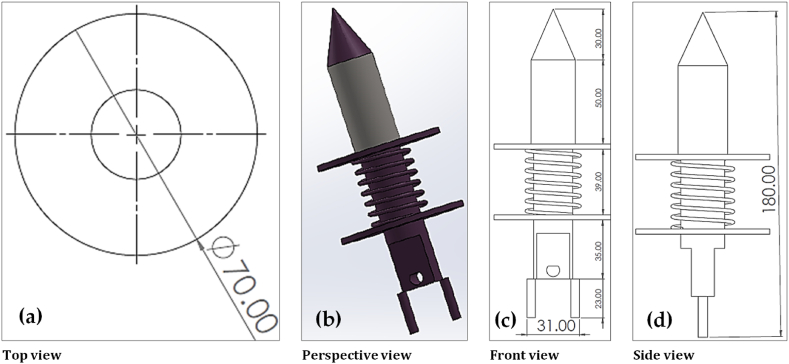
The schematic views of the dibbler: (a) top view; (b) perspective view; (c) front view; and (d) side view. All dimensions are in mm.

**Fig. 5:**
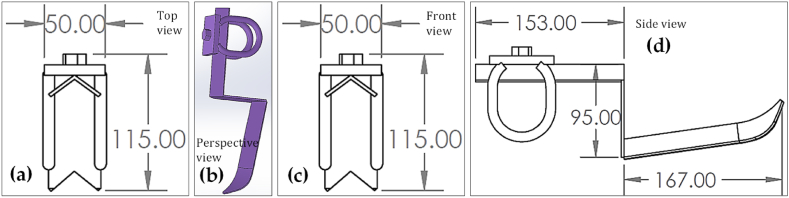
The schematic views of the furrow opener: (a) top view; (b) perspective view; (c) front view; and (d) side view. All dimensions are in mm.

A power tiller operated dibbler vegetable seedling (DVS) and furrow opener vegetable seedling (FVS) transplanters were designed and fabricated at the Farm Machinery and Postharvest Process (FMP) Engineering Division of Bangladesh Agricultural Research Institute (BARI), Gazipur, Bangladesh, during the year 2019–20. The main component of the vegetable seedling transplanter, as shown in Figs. 2 and 3, include a dibbler, chain and sprocket, furrow opener, depth adjusting knob and pressing wheel, in which the dibbler and furrow opener are the main principal parts of the DVS and FVS transplanter, respectively. Most parts of the transplanter are made of ASTM A36 steel, except part of the pressing sprocket, small sprocket and bearing are made of mild steel. However, the specifics and measurements of the various components of the transplanter, together with the standard views of those components, are shown in Figs. 4 and 5, as well as Table 2 and Table 3. A weight-adjusting wheel was included for both transplanters to keep the machine weight and balance in line.

**Table 2:** Specifications of other essential parts of the DVS transplanter.

Name	Schematic diagram	Dimension and works
Pressing sprocket		Diameter- 334 mm Number of teeth-54 Thickness- 13 mm Press the dibbler to make a hole Power transfer
Small sprocket		Diameter- 95 mm Number of teeth-14 Thickness-13 mm Help to remove the dibbler from the soil Power transfer
Chain and Sprocket		Used to transfer the power Dibbler set in chain Maintain seedling-to-seedling distance
Pressing wheel		Diameter-200mm Thickness-50 mm Inner diameter-20mm After putting the seedling then, cover the soil and compact
Bearing		UCB Bearing- P 206 & P 205 supporting the shaft Power transfer

**Table 3:** Specifications of other essential parts of the FVS transplanter.

Name	Schematic diagram	Dimension and works
Ring type Pressing Wheel		Diameter-200mm Thickness-5 mm After putting the seedling then, cover the soil and compact
Metering ruler		Length- 40 cm Width- 5 cm Thickness-5 mm The metering ruler is very useful for accurately placing seedlings in the furrow opener.

In the DVS transplanter design, the larger sprocket was moved to improve the precision of hole-making, where seedlings will be transplanted. To do this, a bigger sprocket was used to press the dibbler into the soil to create the hole. The sprocket was first placed on the transplanter's front side. However, it was then realized that when the pressure is released (after being pressed into the soil), the dibbler returns slightly with an angle resulting in breaking the hole wall and dropping some soil into the hole. So, the larger sprocket was moved to the back to improve the precision hole-making when seedlings are transplanted.

The assembly drawing of the DVS transplanter is represented in Fig. 2. The DVS transplanter has a dibbler, chain, sprocket, seats, press wheel, depth-adjustment wheel, and a hitching point. The power tiller's hitching point, which consists of four nuts and bolts, is where the vegetable transplanter is connected.

Fig. 3 shows the FVS transplanter. Although it looks like a DVS transplanter; however, it plants the seedling differently. In the DVS planter, the seedling is planted in a hole dug by the dibbler, while the FVS transplanter continues to generate furrow, the seedling is placed in the furrow at a distance of 5.08 cm from the furrow opener wall, where the seedling-to-seedling distance in a furrow can be adjusted depending on the requirement of crop seedling. As a result, some soil fills the furrow while covering the seedling's root. The soil is then filled into the furrow and root length of the seedling using a ring-type pressing wheel which also compacts the soil. Dibblers and furrow openers are the fundamental differences between DVS and FVS transplanters. As long as the operators monitor the meter ruler while using the furrow opener, the operators should be able to maintain the distance between the seedlings. The transplanters were designed to plant seedlings at the correct depth without missing them. However, to improve usability and increase the field capacity of the proposed planters, stress analysis was conducted using Solidworks™ software. The parts mainly analyzed were the furrow opener, dibbler, and vegetable transplanter frame (for safety reasons). This analysis used the Huber-Mises-Hencky theory to minimize stress and displacement [19].

#### 2.3.1 Working principle and detail design of DVS transplanter parts

In order to function properly, the DVS transplanter needs to be coupled with a power tiller. The transplanter's chain and sprocket are attached to a wheel shaft, so when the power tiller advances, the DVS transplanter must also advance in the same direction. Power is transferred from the power tiller's wheel shaft to the transplanter's larger sprocket shaft through chain and sprocket. Changing the number of teeth on the larger sprocket also results in a 1.81× increase in travel (as both the power tiller wheel and the sprocket include a secondary, smaller sprocket to keep their forward speeds consistent). For transplanting the vegetable seedlings, two people are required (one for each row). The dibbler is the most vital part of the DVS transplanter; it has a flange, a spring, and a beat point (Fig. 4). It is fastened to the chain, and a hole in the soil can be created with it by means of its use. The chain is connected to two sprockets, one large and one small. The large sprocket exerts downward force on the dibbler, driving the beat point into the soil. The dibbler's flange is used to keep the hole's depth constant. After digging a hole in the soil, spring relieves the pressure. After a seedling has been placed, the soil is covered and compacted using press wheels.

The other parts of the DVS transplanter and their corresponding dimensions are presented in Table 2. A chain is typically described using its grade. The grade of the chain refers to the tensile strength it possesses. There are two sprockets for a chain with a sixty grade; one has 54 teeth, and the other has 14. The average tensile strength of sixty grade chain is 8500 pounds. A larger sprocket is responsible for pushing the dibbler, while the second one is needed to slow down the speed of the dibbler for easy transplant into the hole. The dibbler mounted on a chain is movable to accommodate any distance between the seedlings. The press wheel adjustment enables the operator to modify the press wheel pressure. To provide additional soil cover to the seedling root, both pressing wheels are placed at a 30° angle to the soil surface.

#### 2.3.2 Working principle and detail design of FVS transplanter parts

The working principle of the FVS transplanter is like that of the DVS transplanter; nevertheless, the seedling planting technique is the key difference from the DVS transplanter. Instead of using a dibbler to dig a hole in the soil for seedling implantation, the FVS transplanter employed a furrow opener. The furrow opener is made of iron. The following four factors were considered while designing the furrow opener (i) distance between the soil surface and the transplanter's lower body surface height, (ii) seedling depth, (iii) seedling to seedling distance, and (iv) provide a way for the seedling to be transplanted between the two sides of the furrow opener. The lower portion of the furrow opener is inclined by 10° with a vertical axis for simple seedling placement. The schematic views and detailed dimensions of the furrow opener are shown in Fig. 5.

The other parts of the FVS transplanter and their corresponding measurements are presented in Table 3. A ring-type press wheel was added to the design to compact the soil. The distance between seedlings varies with the crops. Therefore, a metering ruler was also added to the design to place the seedlings in the furrow opener accurately.

### 2.4 Construction of vegetable seedling transplanter

**Fig. 6:**
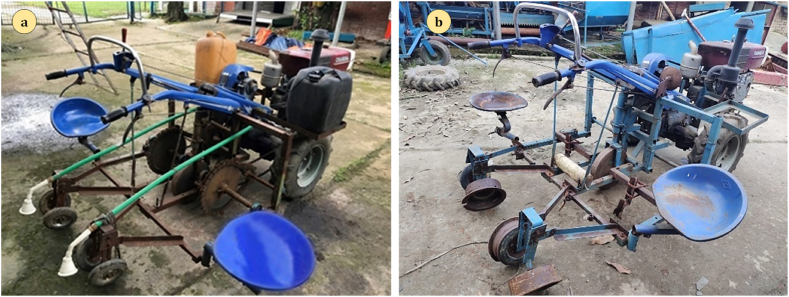
The pictorial view of manufactured: (a) DVS transplanter, and (b) FVS transplanter.

After the CAD design and computer-aided engineering (CAE) procedures, the proposed design were constructed using locally available materials as presented in Fig. 6 (a) and **(b)** for the DVS and FVS transplanters, respectively. Both transplanters have an overall dimension of 3350 × 2000 × 1500 mm. In order to provide water after transplanting, an irrigation system was also attached to both the DVS and FVS transplanters. The approximate construction cost for the DVS and FVS transplanters are 35000 Tk (US$ 345) and 25000 Tk (US$ 245), respectively, costs without power tiller.

### 2.5 Laboratory performance evaluation parameters

A laboratory test was performed to analyze the performances of the designed transplanters. The research test bin at BARI's FMP Engineering Division in Gazipur, Bangladesh was used to perform lab tests and evaluations on the developed transplanters. The missing seedlings, the hole covering, and the field capacity are the parameters that are tested in the lab test. For this experiment, a sandy clay loam soil was used; it had a bulk density of 1420 kg m^−3^ and a field capacity of 30.12%. It is equipped with everything needed to do tests on a variety of parameters, and it uses established scientific methods for determining the results [32]. The experimental equipments was used in the study. In order to avoid inaccuracy and tolerance for laboratory and field experimental equipments, there are a number of variables that must be followed in order to obtain measurements that are more accurate [33]. These variables include precise equipment selection, the unit being tested, an efficient operator, environmental factors, and accurate methods [34,35].

#### 2.5.1 Missing seedlings

In the experiments, missing seedlings were defined as the result of extraction failure or insufficient gripping between finger and growing media, and it was formulated as given by Equation (1):

(1) MS(%)=NMSx100/NSF

where, NMS is the number of missing seedlings, MS is the missing seedlings (%), and NSF is the number of seedlings fed.

#### 2.5.2 Hole covering

As soil moisture is an essential factor in sustaining and standing seedlings, the performance of the hole covering is an essential component to consider when assessing the effectiveness of the transplanter. If the seedling is exposed to the air or uncovered, the water evaporates, and the plant cannot uptake the soil water along with its nutrients. Consequently, soil coverage is crucial to covering performance. The percentage of soil covered was calculated by determining how much soil remained in the hole using gravimetric analysis just after the seedling was planted. Ten locations over each plot were chosen randomly, and the remaining loose soil was collected using a wooden scoop and fiber brush. The samples were dried in an oven at 105 °C for 48 h to get their dry mass [36]. The sand replacement method was used to identify the volume of the corresponding hole. Equation (2) was used in order to determine the percent hole covering [24].

(2) $$Hole\;covering=\left(W_{d}/V_{Q}\right)\times 100$$

where, V_Q_ is the volume of the hole (m^3^), and W_d_ is the weight (kg) of the remaining dry soil.

#### 2.5.3 Field capacity

The types of field capacity (FC) are theoretical field capacity (TFC) and effective field capacity (EFC). The TFC is the actual rate of field coverage achieved in a given amount of time while the transplanter is in operation. When the transplanter is in operation, EFC may be defined as the actual field rate that occurs within a certain amount of time. It is to be noted that the forward speed and effective width are the same for both types of transplanter. The study aims to increase seedling efficiency compared to manual transplanting. The TFC and EFC was calculated by Equation (3), and Equation (4), respectively [24].

(3) $$TFC\;\left(ha\;h^{-1}\right)=S\cdot W/10$$

where, W represents the width of coverage (m), and S represents the forward speed (km h^−1^).

(4) $$EFC\;\left(ha\;h^{-1}\right)=Actual\;field\;coverage\;\left(ha\right)\;/\;Actual\;time\;of\;operation\;\left(h\right)$$

The field efficiency was calculated by Equation (5) based on the TFC and EFC.

(5) Fieldefficiency(%)=(TFC/EFC)x100

### 2.6 Field experiments setup

During the rabi season (winter) of 2019-20 and 2020–21, field experiments were carried out in the FMP Engineering Divisional research field located at BARI, Gazipur. The designed transplanters were tested with the brinjal crop. The treatments adopted in this study were defined as T_1_, which involved manual transplantation; T_2_, which involved seedling transplantation using a DVS transplanter; and T_3_, which involved seedling transplantation using an FVS transplanter. The experiments were conducted out using a randomized complete block design with four distinct replications. The fertilizer application method was done by broadcasting 10 DAT (days after transplanting) and 50 DAT for brinjal (which is the common practice in Bangladesh). To avoid the equipment measurement errors, the environmental factors in the atmosphere like temperature, humidity, pressure, gravity, elevation, stress, strain, and lighting have been adjusted when field experiments were performed. A failure to account for the effect of the environment not only contributes to the uncertainty, but it also has the potential to result in additional measurement error [37].

**Fig. 7:**
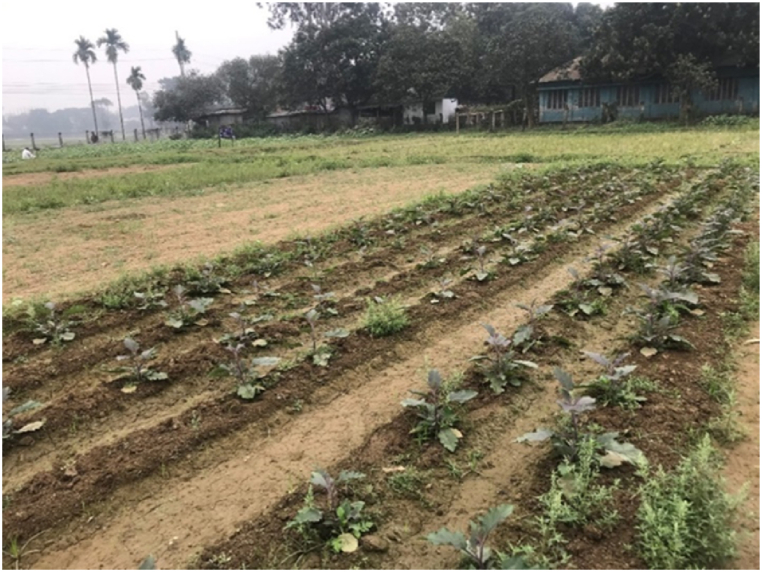
Pictorial view of the experiment field.

Before field experiment testing, the soil surface was leveled after plowing, performed using a 110-horsepower MF399 tractor at 25 cm depth. The detailed agronomic practices with fertilizer and irrigation requirements for the brinjal crop were followed as the handbook prescribes [38]. Because the transplanter was only equipped with a single cultivating mechanism, the seedlings were laid out in two rows during planting. The seedlings used were in the four leaves stage at the transplanter time. Keep in mind, vegetable transplantation should only be done in the evening since vegetable crops are responsive to variations in weather and need immediate operations, this gives plants time to adjust to the cool nighttime temperature and recuperate from the shock of transplanting before the following day. In addition, after removing the plants from the nursery, the transplanting procedure should be completed as quickly as possible. Details of the field experiment are given in Table 4, and the experimental field view is presented in Fig. 7.

**Table 4:** Details of the field experiment for brinjal crops.

SL. No.	Parameters	Brinjal
1	Variety	BARI Begon-8
2	Plot size (m^2^)	1.5 × 20
3	Line to line (m)	0.7
4	Plant to plant (m)	0.6

### 2.7 Data collection and analysis

The laboratory tests evaluated the data for the paramenters of the depth of placement, the distance between seedling to seedling, row-to-row distance, the percentage of hole covering, and missing placement. Data gathering for the field experiments followed the standard agronomic field experiment procedures adopted from the protocol [39]. The performance of the developed transplanters were evaluated using field trials. These studies tracked metrics including field capacity, field efficiency, and the amount of time the transplanters saved during operations. Based on the total number of plants per plot, crop productivity and yield-contributing characteristics (such as plant height, fruit height, length, and weight) data were also collected. R, an open-source statistical computing program created by the R Foundation for Statistical Computation, was used for the statistical study [40]. Significant changes (α = 0.05) between treatments were identified using analysis of variance (ANOVA) testing. In order to compare the gathered data, methodologies for the Duncan's Multiple Range Test, often known as the DMRT, were developed and LSD was used to compare the means (with a 5% probability).

## 3 Results and discussion

### 3.1 Design analysis results

**Fig. 8:**
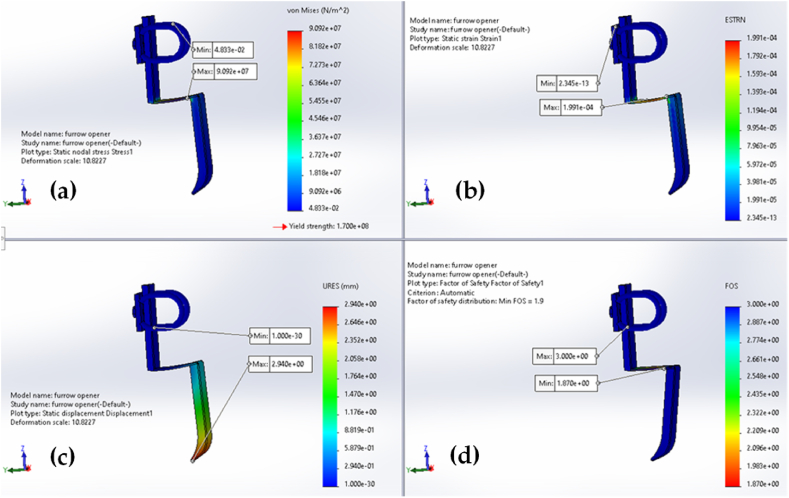
Furrow opener analyses with application of 100 N force for: (a) stress; (b) strain; (c) displacement; and (d) factor of safety.

**Fig. 9:**
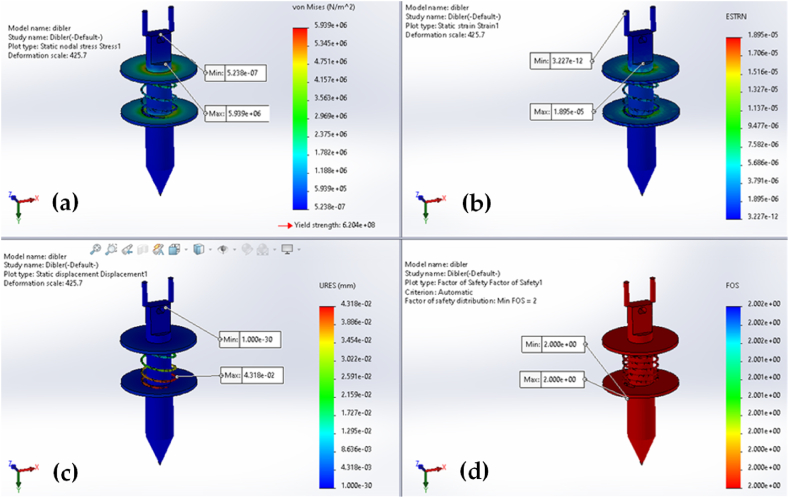
Dibber analyses with application of 200 N force for: (a) stress; (b) strain; (c) displacement; and (d) factor of safety.

**Fig. 10:**
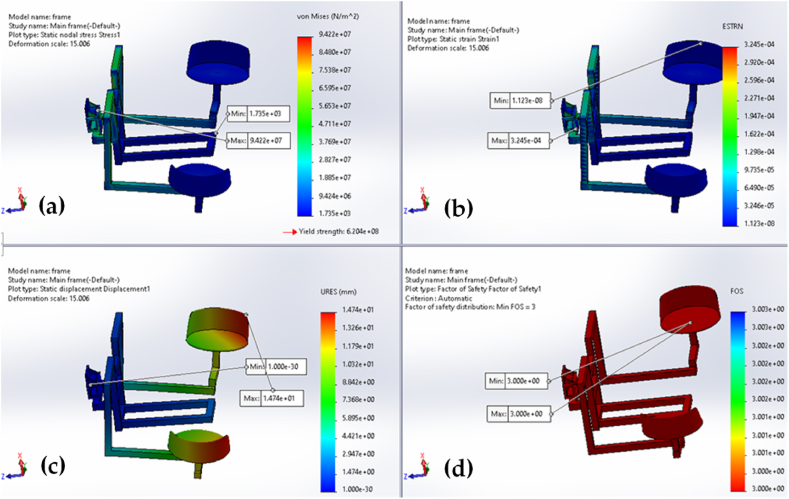
Transplanter frame analyses with application of 2500 N force for: (a) stress; (b) strain; (c) displacement; and (d) factor of safety.

The design analysis results for the furrow opener, dibbler, and frames of vegetable seedling transplanters are shown in Fig. 8, Fig. 9, and Fig. 10, respectively. The magnitudes that are represented by the colors red, green, and blue in the figures are the highest, medium, and lowest, respectively. The design analyses were carried out based on the load imposed on the specific parts of the transplanter. To do so, 100 N, 200 N, and 2500 N forces were considered for the furrow opener, dibbler, and frame, respectively (as these are the maximum forces exerted on the corresponding parts) [24]. It is important to keep in mind that a factor of safety value of 1.0 indicates that the material is starting to distort in a particular place.

The factor of safety distribution is shown in Fig. 8, and it can be seen that the lowest factor of safety (FOS) was 1.9. This indicates that the material would start to yield if the force almost doubled. The results also showed that the furrow opener yield strength was 1.70 × 10^8^ Nm^−2^, suggesting that the covering part of the furrow opener will start to deflect in the other direction when the magnitude of the strength is greater than this amount (Fig. 8). It was observed that this material had begun to yield when the von Mises stress approached the yield strength of 1.70 × 108 Nm^−2^.

The highest stress, strain, and displacement were 9.09 × 10^7^ Nm^−2^, 1.99 × 10^−4^, and 2.94 mm, respectively. On the other end of the spectrum, the lowest stress, strain, and displacement were 4.83 × 10^−2^ Nm^−2^, 2.34 × 10^−4^, and 1.00 × 10^−3^ mm, respectively. It was revealed that the point of connection at the horizontal flat bar and lower part of the furrow opener was the location that experienced the greatest amount of stress and strain, and that this stress and strain gradually decreased as one moved from the center to the left or right side. Additionally, it was found that the displacement was greatest at the furrow opener's tip points. Note that after 200 h of operation, no breakdown was observed on the machine parts. The same design analysis was also applied to the dibbler and transplanter frame in Figs. 9 and 10, respectively.

### 3.2 Performance test results

#### 3.2.1 Laboratory performance for the DVS and FVS transplanters

Table 5 shows that the average depth of planting was 3.25 cm, and the spacing between seedlings ranged from 25.8 to 62 cm, almost little variation was seen in either value in the DVS transplanter. After the seedling has been placed in the hole, the hole has to be entirely filled up with soil (to about 100%); otherwise, the seedling might suffer and die as a result of excessive loss of water [41]. According to the results, the average hole coverage was found to be nearly 115%. Additional soil is given for earthing-up after the hole has been covered; this was done by pressing wheel. The effect of pressing wheel angle on plant standing was found significant. The tilt angle of the pressing wheel for the corresponding hole coverage by soil after seedling placement in the soil hole needs to be optimized to get 100% plant stand in the vertical direction. For this, the tilt angle could always be maintained in this study as 15%, as this inclination angle has been considered an optimum angle [12,42]. The width of the pressing wheel (200 mm) has a significantly positive effect on plant standing, and this simultaneous action of tilt inclination angle and width of the pressing wheel has played a pivotal role in the first stage of seedling standing. Results also show that no seedlings were missing in the transplanting procedure. However, it was determined from the laboratory experiments that maintaining row-to-row distance is difficult when a DVS transplanter is used as there is a considerable variation between the minimum and maximum row-to-row distance (from 50 to 81 cm).

It was evident from Table 5 that the average seedling inclination was 19.5°, which means the transplanter performance is in good agreement with regard to the seedling placement and standing, as evident from the studies reported in the control laboratory experiment [12].

**Table 5:** Performance of DVS transplanter at laboratory test.

Parameters	Unit	Observations										Mean
		1	2	3	4	5	6	7	8	9	10	
Depth of placement	cm	3.1	3.24	3.46	2.85	3.66	3.5	3.41	3.0	3.0	3.32	3.25
Distance between seedling to seedling	Min. (cm)	25	23	29	28	26	25	28	24	26	24	25.8
	Max. (cm)	61	63	60	62	62	63	62	62	61	63	62
Row-to-row distance	Min. (cm)	50	50	50	50	50	50	50	50	50	50	50
	Max. (cm)	81	81	81	81	81	81	81	81	81	81	81
Tilt angle of pressing wheel	Degree	30	30	30	30	30	30	30	30	30	30	30
% of hole covering	–	120	115	108	125	121	109	110	117	115	115	115.5
Missing of placement	–	0	0	0	0	0	0	0	0	0	0	0
Seedling inclination	Degree	23	12	15	24	19	25	26	18	12	22	19.5

Note: Min. = minimum, max. = maximum.

The results of the FVS transplanter tested in the laboratory are given in Table 6. Results showed that the furrow opener could maintain seedling roots between 2 and 8 cm in depth, depending on the seedling. The average minimum distance between seedlings was measured as 12.9 cm, while no variation for maximum distance between seedlings was detected as the furrow opener draws the line at a prescribed depth (so any distance between seedling to seedling can be maintained). The average hole coverage for plant standing was measured as 118.2%, which is nearly the standard value, as evident from the studies reported [12,42]. And no missing seeding placement was observed during the lab test. It is obvious that there was no statistically significant difference between the effects of the two transplanting methods on plant standing. The minimum and maximum row-to-row distances were maintained at 20 and 80 cm, respectively. Here noted that backfill is not a significant issue with FVS transplanters because the seedling was planted about 2 inches from the furrow opener's wall, so some soil fills in the furrow and covers the seedling's root [[42], [43], [44]]. The average seedling inclination was measured as 12.8°, which is also supported by the study [12]. Here also noted that seedling placement was not missing for both transplanters when the operator of the seedling placement fed the seedling at a forward speed of 1.2 km h^−1^. Therefore, it can be said that the effects of seedling transplanting and standing for both transplanters have been significantly positive with any seedling size.

**Table 6:** Performance of FVS transplanter at the laboratory test.

Parameters	Unit	Observations											Mean
		1		2	3	4	5	6	7	8	9	10	
Depth of placement		2–8 cm depth of the seedling root											
Distance between seedling to seedling	Min. (cm)	11	13		14	15	12	13	11	13	13	14	12.9
	Max. (cm)	Any distance can maintain according to the crop											any
Row-to-row distance	Min. (cm)	20	20		20	20	20	20	20	20	20	20	20
	Max. (cm)	80	80		80	80	80	80	80	80	80	80	80
Tilt angle of pressing wheel	Degree	30	30		30	30	30	30	30	30	30	30	30
% of hole covering		119	118		118	119	121	117	118	117	116	119	118.2
Missing of placement		0	0		0	0	0	0	0	0	0	0	0
Seedling inclination	degree	8	12		11	14	12	11	16	14	11	19	12.8

Note: Min. = minimum, max. = maximum.

#### 3.2.2 Field experiment performance for yield productivity

The results from the field performance test, including the field capacity, field efficiency, and the amount of operational time saved by the transplanters, are shown in Table 7. It shows that the effective field capacity (EFC) of the FVS and DVS transplanters is higher than that of the manual transplantation treatment (0.05 vs. 0.039 ha h^−1^). Both transplanter's field efficiency was 61.18%, higher than the manual transplantation treatment and the other manual transplanting methods reported in the literature [18]. Additionally, the results demonstrated that, compared to manual transplanting, the FVS and DVS transplanters could complete seedling transplantation 21.32% more quickly. The results also show that the effects of forward speed of power tiller and operator's seedling transplanting on missing plants were shown to be significant with a 5% level of significance for both transplanters and manual transplanting methods, as evident from Table 4, Table 5, and Table 6. The missing of seedling planting by the operators was found as zero and the seedling inclination angle for vertical standing was found to be close to 15°, it is clear that they can easily feed the seedling. It should also be noted that the EFC and field efficiency values depend on the transplanted vegetable seedling. Overall, the results suggest that the FVS and DVS transplanters can be good alternatives for their multiple positive benefits towards adopting seedling transplanting into the subsoil applications.

**Table 7:** Evaluating the field performance of vegetable transplanters with regard to effective field capacity, field efficiency, and the amount of time saved by the transplanters for the brinjal crop.

Treatment	Forward Speed (km/h)	Effective width (m)	Theoretical field capacity (ha/h)	Time required (h/ha)	Time saved (%)	Effective field capacity (ha/h)	Effective field capacity (dec/h)	Field efficiency (%)
T_1_	1.05	0.681	0.072	25.42	0.00	0.039 b	9.72 b	55.02 b
T_2_	1.2	0.681	0.082	20.00	21.32	0.05 a	12.35 a	61.18 a
T_3_	1.2	0.681	0.082	20.00	21.32	0.05 a	12.35 a	61.18 a
Mean	1.15	0.68	0.08	21.81	–	0.05	11.47	59.13
CV (%)	7.53	–	7.53	–	–	13.25	13.25	6.02
SD	0.09	–	0.01	–	–	0.01	1.52	3.56
LSD (0.05)	–	–	–	–	–	*	*	*

Note: T_1_-manual transplanting; T_2_-seedling transplanting by DVS transplanter; T_3_-seedling transplanting by furrow FVS transplanter. * - Significant with a 5% level of significance.

Table 8 displays the results of brinjal crop yield tests conducted using different treatments. According to the findings, crop yields were significantly higher in treatments in which a transplanter was used (P 0.05) than the manual transplanting. Results also revealed that higher yields were observed for the T_3_ compared to the T_1_ and T_2_ (P < 0.05), while the T_1_ was found as the least effective treatment. In Bangladesh, the average yield of brinjal is 20–24 t ha^−1^ [45], whereas the findings for DVS and FVS transplanters were 25.95 and 26.26 t ha^−1^, respectively. The highest yield in vegetable seedling transplanter can be attributed to the soil loosening capability of the FVS transplanter, which was done by furrow opener and pressing wheel. The furrow opener makes a narrow furrow that is wide enough to accommodate the seedling to be transplanted and wide enough to allow the soil to move on both sides of the furrow. After the seedlings have been transplanted, the narrow furrow is filled from both sides with the help of a set of pressing wheels designed for covering [46]. For both FVS and DVS transplanters, the field performance data is nearly identical to average yield data, with no statistical difference, which shows that the soil covered around the seedlings is good enough in both transplanters and maintains the proper depth for all the seedlings, resulting in the uptake of nutrients from the soil, allowing the soil nutrients to be available in the crop root zone for a longer period of time [10,47]. Therefore, it is evident from the results that the transplant of vegetable seedlings by the transplanter positively impacts increasing crop yield. In addition, the yield-contributing properties of brinjal crops grown under a variety of treatments are shown in Table 8. Most of the yield contributing parameters varied significantly at 5% level. However, most yield-contributing characteristics showed the highest value in treatment T_3,_ which was identical to the treatments of T_2_ and T_3_ [48,49]. For both transplanters, the field performance data is nearly identical to the average yield data, with no statistical difference in the yield.

**Table 8:** Yield and yield contribution parameters of Brinjal crop.

Treatment	Plant height (cm)	Fruit number /plant	Fruit length (cm)	Fruit weight (avg. of five fruits) (g)	Yield (t ha^−1^)
T_1_	98.0 b	13.7 c	29.3 b	367.3 c	25.28 c
T_2_	101.13 a	13.9 b	29.7 a	377.5 b	25.95 b
T_3_	101.29 a	14.2 a	29.8 a	379.5 a	26.26 a
Mean	100.14	13.93	29.60	374.77	25.83
CV (%)	1.85	1.81	0.89	1.75	2.11
LSD (0.05)	*	*	*	*	*

Note: T_1_-manual transplanting; T_2_-seedling transplanting by DVS transplanter; T_3_-seedling transplanting by FVS transplanter. * - Significant with a 5% level of significance.

## 4 Study limitations

Mechanized seedling transplanting of tomato, eggplant, cabbage, cauliflower and chili, among others, in combination with other improved crop production practices, can increase crop yield and quality in Bangladesh, as manual transplanting is a time-consuming, labor-intensive, non-uniform distribution of seedling and expensive operation that also causes muscular fatigue to the operator due to prolonged squatting posture [50].

The semi-automatic vegetable transplanters are challenging to run because there are requirements for manual seedling feeding. These constraints are variable and rely on the amount of time spent working as well as the skill of the operator. In addition, the process of seedling feeding to the delivery unit is challenging due to singulation, selection, alignment and manual transfer of seedlings by the operators. In the DVS and FVS transplanters, a separate feeding system has not been developed, and the feeding mechanism provision has also not been incorporated. It has to be a great opportunity to draw and establish the separate seedling feeding mechanism, which might be powered from the gearbox of the power tiller or other means of wheel to power the feeding mechanism to complete the automatic transplanter. For this, this semi-automatic seedling transplanter needs to add the feeding tray to convert it to the automatic seedling transplanter.

## 5 Conclusions

The mechanized vegetable seedling transplanter has shown significant potential in improving seedling efficiency and providing value in agricultural vegetable production practices. The development and testing of power tiller-operated vegetable seedling transplanters (FVS and DVS) specifically cater to the needs of small to medium-level farmers, aiming to enhance the efficiency of transplanting vegetable seedlings. The primary design factors, the dibbler and the furrow opener, have been successfully incorporated into the DVS and FVS transplanters, respectively. The dibbler, connected to the larger sprocket by a chain, exerts a downward force when pushed into the soil results in the creation of a hole for seedling transplantation. Conversely, a furrow opener is employed instead of a dibbler, which generates furrows for the placement of seedlings. The results of our study have shown that the DVS and FVS transplanters allow for adjustable spacing between row-to-row, ranging from 50–81 cm and 20–80 cm, respectively. Similarly, seedling to seedling can be adjusted from 25.80–62 cm and 12.5 cm to any distance for the DVS and FVS transplanters, respectively. Importantly, both types of transplanters exhibited precise seedling placement without any instances of missing seedlings during laboratory test. Comparatively, the FVS transplanter demonstrated a lower degree of vertical axis inclination after transplanting, highlighting its superior performance than the DVS transplanter. The average depth of placement for DVS transplanters can be maintained at 3.25 cm, while the FVS transplanter allowed for a wider depth range of 2–8 cm. Field experiments conducted at an average forward speed of 1.2 km h^−1^ revealed notable field capacity and field efficiency, with value of 0.05 ha h^-1^ and 61.18% for the DVS and FVS transplanters, respectively. Furthermore, the findings of the field experiment also demonstrated that the parameters related to brinjal yield and yield contribution remained statistically unchanged, confirming the viability of the transplanter for practical use. In summary, semi-automatic mechanized vegetable seedling transplanter offers a range of benefits, including reduced labor, time savings, and the ability to ensure an equitable distribution of seedlings. These advantages make it an attractive option for small to medium-level farmers seeking to engage in semi-automated vegetable farming practices. By embracing this technology, farmers can enhance their productivity and efficiency while contributing to sustainable agricultural practices.

## Funding statement

This work was supported by Researchers Supporting Project number (RSP2023R100), 10.13039/501100002383King Saud University, Riyadh, Saudi Arabia. This research study was funded by the core research program of the 10.13039/501100005867Bangladesh Agricultural Research Institute.

## Author contribution statement

Md Sumon Miah: Conceived and designed the experiments; Performed the experiments; Analyzed and interpreted the data; Contributed reagents, materials, analysis tools or data; Wrote the paper.

Md Mashiur Rahman: Performed the experiments; Analyzed and interpreted the data; Contributed reagents, materials, analysis tools or data; Wrote the paper.

Muhammad Arshadul Hoque: Conceived and designed the experiments; Performed the experiments; Analyzed and interpreted the data.

Sobhy M. Ibrahim, Muhammad Sultan and Redmond R. Shamshiri: Analyzed and interpreted the data; Wrote the paper.

Mustafa Ucgul, Mahedi Hasan and Tasneem Nahar Barna: Analyzed and interpreted the data; Contributed reagents, materials, analysis tools or data; Wrote the paper.

## Data availability statement

Data included in article/supp. material/referenced in article.

## Additional information

No additional information is available for this paper.

## Declaration of competing interest

The authors declare that they have no known competing financial interests or personal relationships that could have appeared to influence the work reported in this paper.
